# Oral histoplasmosis

**DOI:** 10.4103/0972-124X.60230

**Published:** 2009

**Authors:** Karthikeya Patil, V. G. Mahima, R. M. Prathibha Rani

**Affiliations:** *Department of Oral Medicine and Radiology, JSS Dental College and Hospital, JSS University, Mysore - 15, India*

**Keywords:** Oral histoplasmosis, oral ulcer, AIDS

## Abstract

Histoplasmosis is a systemic fungal disease that takes various clinical forms, among which oral lesions are rare. The disseminated form of the disease that usually occurs in association with Human Immunodeficiency Virus (HIV) is one of the AIDS-defining diseases. Isolated oral histoplasmosis, without systemic involvement, with underlying immunosuppression due to AIDS is very rare. We report one such case of isolated oral histoplasmosis in a HIV-infected patient.

## INTRODUCTION

Histoplasmosis is a granulomatous systemic mycosis caused by the dimorphic fungus *Histoplasma Capsulatum*, the clinical disease of which was first described by Samuel Darling in 1905. Oral histoplasmosis usually occurs in association with the chronic disseminated form of the disease. Sometimes they may present as the initial or the only mucocutaneous manifestation of the disease.

## CASE REPORT

A 45-year-old male patient reported with a chief complaint of painful ulcer on the left side of his tongue since two months. The patient gave no history of trauma prior to the onset of the ulcer. There was a history of associated pain and bleeding from the ulcer with difficulty in speech, mastication, and deglutition, with no history of pus discharge, paresthesia or numbness. Also, there were no systemic symptoms. The medical and personal histories were noncontributory.

**Figure 1:**
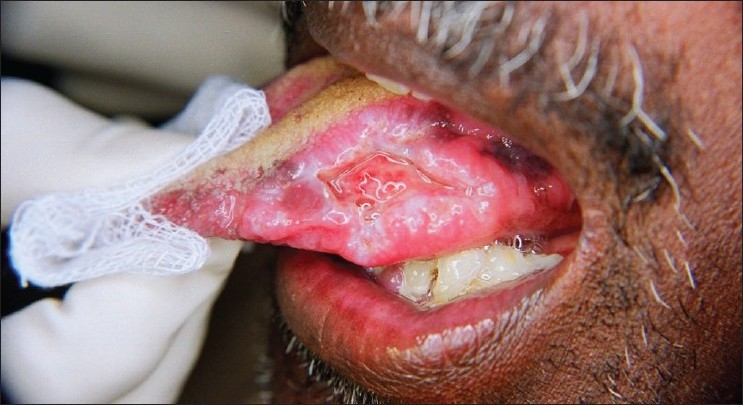
Ulcer present on the left lateral border of the tongue

**Figure 2:**
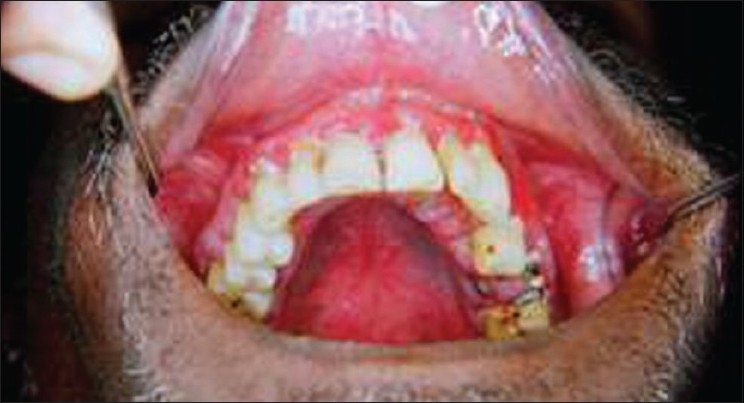
Diffuse granular enlargement of maxillary gingiva

**Figure 3:**
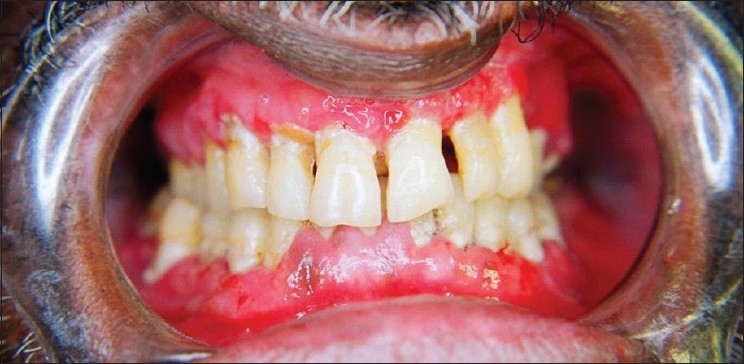
Diffuse granular enlargement of maxillary and mandibular gingiva

An extraoral examination revealed left submandibular lymphadenopathy, and intraoral examination revealed a solitary, large, deep ulcer on the left dorsum of the tongue, which was roughly rhomboidal in shape and measured 1.0 × 2.5 cm. The borders of the ulcer were edematous and raised, the floor appeared granular, and was covered with slough [Figure 1]. The ulcer was tender and indurated on palpation. Diffuse granular enlargement of marginal, interdental, and attached gingiva of the maxilla and mandible was conspicuous, with focal areas of necrosis [Figures 2 and 3].

**Figure 4:**
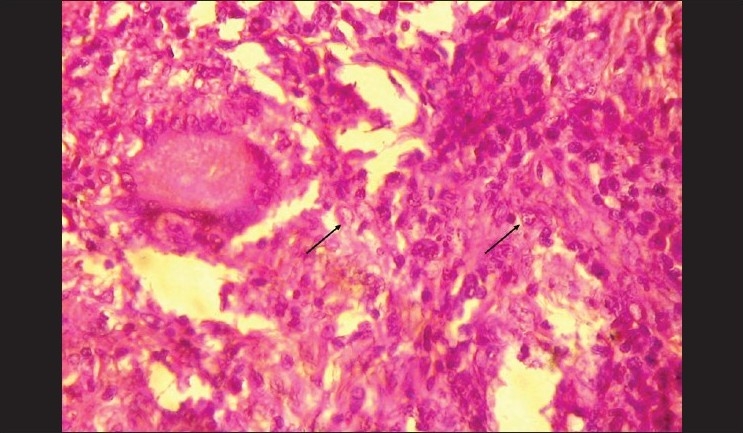
H and E, ×10 section showing langhans giant cells, epithelioid cells, and foamy macrophages

**Figure 5:**
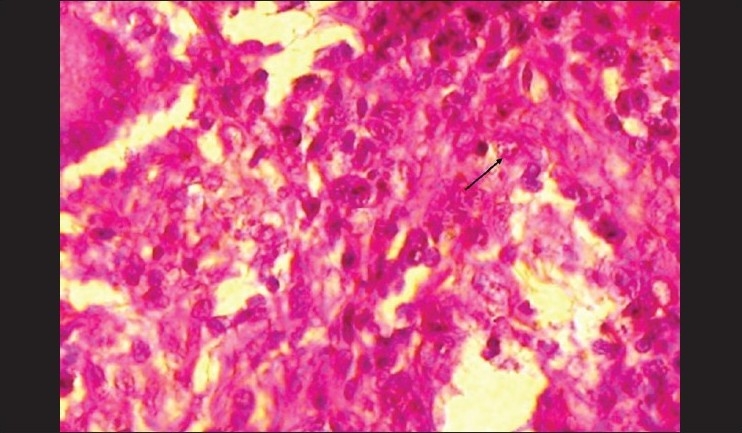
H and E, ×40 showing *Histoplasma Capsulatum* organisms within macrophages. No evidence of caseous necrosis or malignancy noted.

A panoramic radiograph revealed generalized, moderate, horizontal bone loss. A chest radiograph did not reveal any abnormality. The patient was reactive for HIV 1 and 2 using Western blot assay. The fungal culture of the tissue from the tongue and left maxillary palatal gingiva showed the presence of normal oral flora and no evidence of mycobacterium tuberculi. The histopathologic sections revealed an ulcerated, stratified, squamous epithelium in relation to the tongue and an intact epithelium in relation to the gingiva, with dense chronic inflammation, numerous epitheloid cell granulomas, few Langhans-type giant cells, and foamy macrophages [Figure 4]. The foamy macrophages showed numerous small fungal spores surrounded by a clear halo. There was no evidence of caseous necrosis or malignancy in the tissue sections.[Figure 5]. The histological features were suggestive of histoplasmosis. Based on the clinical features and investigatory findings, a final diagnosis of localized oral histoplasmosis in a HIV seropostive patient was made. Further, the patient was referred to a general physician for complete systemic evaluation, where systemic involvement of histoplasmosis was ruled out.

**Figure 6:**
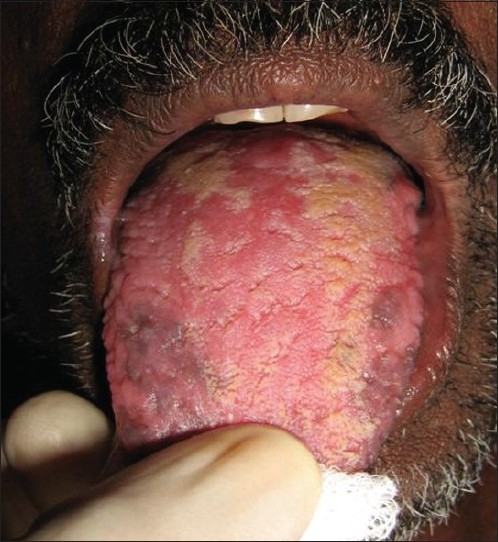
Post treatment showing complete resolution of tongue lesion

**Figure 7:**
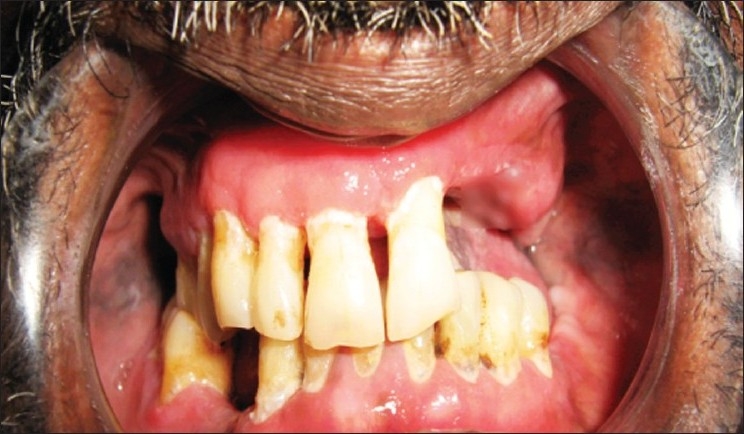
Post treatment showing complete resolution of gingival lesion

After counseling the patient, anti-retroviral therapy was instituted as a part of the general management. T. Itraconozole 200 mg TID for three days followed by T. Itraconozole 200 mg BD for 12 weeks was instituted, as a part of a specific therapy. The lesions completely healed after a complete course of medication [Figures 6 and 7]. The patient, however, is under regular follow-up and no recurrence is noted.

## DISCUSSION

*Histoplasma Capsulatum* is a dimorphic fungus that assumes a yeast form, about 1-4 microns in diameter, in the host tissue. It is found chiefly in warm, humid environment that contains bird and bat excreta, and soil high in nitrogen content.[1–3] Although *Histoplasma Capsulatum* is endemic, sporadic cases have been reported throughout the world. Humidity and soil characters[2,3] have been attributed for its endemic distribution. Padhye *et al*., suggested that histoplasmosis in Indians tends to occur primarily in the extrapulmonary sites, particularly in the oral cavity.[2] Clinically it can take an acute pulmonary, a chronic pulmonary or a disseminated form.[4]

Oral lesions of the disease manifests rarely. When present, they occur in association with the disseminated form or sometimes as a localized lesion, which could be the initial[2,5] or the only manifestation of the disease.[2–6] They are common in men, with a male to female ratio of 9:1.[1–8] The mean age of occurrence is 39 years, with an average ranging from 26 to 65 years. The commonly involved sites in the oral cavity are tongue, hard and soft palate, buccal mucosa, gingiva, and lips.[1–5] According to Goodwin *et al*., the oropharyngeal lesions are frequently the initial presentation of the disease, especially in the disseminated form.[2] Reddy *et al*. (1970), reported that all the patients who presented initially with oral lesions, subsequently developed disseminated disease,[2] suggesting the need for a periodic evaluation of patients with localized oral histoplasmosis, for any systemic involvement. Oral lesions can manifest as papular, ulcerative, nodular, vegetative, furunculoid, granulomatous, or plaque-like lesions, with the most common presentation being a shallow or deep infiltrated ulceration with a pseudomembrane.[1–5,9,10] Gingival manifestations include ulcerative and painful granulomatous lesions.[8] Sore throat, hoarseness of voice, and dysphagia can also manifest.[3]

Histoplasmosis can be diagnosed based on clinical signs and symptoms, histopathology, cultures, serologic test, including compliment fixation test, immunodiffusion, and histoplasmin skin test. Diagnosis by fungal culture provides the strongest evidence of infection, but that is useful in progressive, disseminated, or chronic pulmonary histoplasmosis, rather than in the initial cases. Care should be taken not to exclude the diagnosis in false negative cases.[7] Histopathology is the prime investigative modality, as identification of Histoplasma organism in the sections provides conclusive evidence of the disease.[4] Serologic tests have limited value in HIV patients because of diminished antibody production. Histoplasmin skin test is of limited value, as the reagents are no longer available. Additionally, a positive histoplasmin skin test boosts antibody levels, compromising the interpretation of serologic tests.[7] Direct immunofluorescence is diagnostic in case of HIV patients.[7]

The disease is self-limiting in immunocompetent patients.[3,4] Amphotercin B, at a dose of 2 gms IV for 10 weeks, is used in the management of pulmonary histoplasmosis, in HIV patients. Studies have shown that in immunocompetent patients without AIDS, amphotercin B is effective by 68-92%, itraconozole by 100%, and ketaconozole by 56-70%, whereas, in patients with AIDS, amphotercin B is effective by 74-88% and itraconozole by 85%.[3] In addition, itraconozole is known to have rapid action and is effective in preventing a relapse.[8]

## CONCLUSION

The consistently raising incidence of histoplasmosis in India and other parts of Asia is quiet alarming. As the disease is the second most common opportunistic infection associated with HIV, and is one of the AIDS defining diseases,[5,8] it should be considered in the differential diagnosis of an unusual, exaggerated, oral ulceration, when encountered with HIV seropositivity. When such cases are encountered, an attempt must be made to evaluate the underlying HIV infection. A thorough clinical knowledge about oral histoplasmosis is important in diagnosing and preventing further dissemination, thereby, preventing the fulmination of this fatal disease in order to improve and prolong the lives of patients with AIDS.
